# Inhibitory Effects of Auraptene and Naringin on Astroglial Activation, Tau Hyperphosphorylation, and Suppression of Neurogenesis in the Hippocampus of Streptozotocin-Induced Hyperglycemic Mice

**DOI:** 10.3390/antiox7080109

**Published:** 2018-08-19

**Authors:** Satoshi Okuyama, Tatsumi Nakashima, Kumi Nakamura, Wakana Shinoka, Maho Kotani, Atsushi Sawamoto, Mitsunari Nakajima, Yoshiko Furukawa

**Affiliations:** Department of Pharmaceutical Pharmacology, College of Pharmaceutical Sciences, Matsuyama University, 4-2 Bunkyo-cho, Matsuyama, Ehime 790-8578, Japan; mu.yakuri.009@gmail.com (T.N.); mu.yakuri.008@gmail.com (K.N.); mu.yakuri.010@gmail.com (W.S.); mu.yakuri.011@gmail.com (M.K.); asawamot@g.matsuyama-u.ac.jp (A.S.); mnakajim@g.matsuyama-u.ac.jp (M.N.); furukawa@g.matsuyama-u.ac.jp (Y.F.)

**Keywords:** auraptene, naringin, hyperglycemia, neurogenesis, tau phosphorylation, anti-inflammation, anti-oxidation

## Abstract

Auraptene, a citrus-related compound, exerts anti-inflammatory effects in peripheral tissues, and we demonstrated these effects in the brains of a lipopolysaccharide-injected systemic inflammation animal model and a brain ischemic mouse model. Naringin, another citrus-related compound, has been shown to exert antioxidant effects in several animal models. Hyperglycemia induces oxidative stress and inflammation and causes extensive damage in the brain; therefore, we herein evaluated the anti-inflammatory and other effects of auraptene and naringin in streptozotocin-induced hyperglycemic mice. Both compounds inhibited astroglial activation and the hyperphosphorylation of tau at 231 of threonine in neurons, and also recovered the suppression of neurogenesis in the dentate gyrus of the hippocampus in hyperglycemic mice. These results suggested that auraptene and naringin have potential effects as neuroprotective agents in the brain.

## 1. Introduction

Recent research shows that hyperglycemia induces inflammation, reactive oxygen species production and neuronal dysfunction in the central nervous system, and those are linked to a number of disorders [1]. Diabetes was recently identified as a risk factor for Alzheimer’s disease (AD) [2,3,4]. The AD brain is frequently associated with severe inflammation, oxidative stress, neuronal dysfunction, amyloid-beta accumulation, tau hyperphosphorylation, and memory impairment [5]. A well-established hyperglycemia diabetes model is induced by streptozotocin (STZ), a glucosamine-nitrosourea compound. STZ induces insulin-secreting pancreatic beta cell death through DNA methylation [6], resulting in chronic hyperglycemia and hypoinsulinemia. Severe oxidative stress, inflammation, tau hyperphosphorylation, and neuronal dysfunction have been observed in the brain of the STZ-induced hyperglycemia model [7,8].

We previously demonstrated that auraptene (AUR), a coumarin compound from citrus fruit, exerted anti-inflammatory and neuroprotective effects in the brain of a lipopolysaccharide (LPS)-injected inflammation model [9]. In this experiment, an intranigral injection of LPS induced microglial activation and dopaminergic neuronal death; on the other hand, an AUR treatment suppressed microglial activation and neuronal cell death. Furthermore, AUR exerted suppressive effects on astrocyte activation and neuronal cell death in the hippocampus of a transient global cerebral ischemic mouse model [10]. Naringin (NGI) is also a biologically active flavonoid substance from citrus that has been shown to exert antioxidant, anti-inflammatory, and neuroprotective effects in the brains of several brain disorder models [11,12,13,14]. Therefore, the aim of the present study was to investigate whether both AUR and NGI show anti-inflammatory and anti-tau hyperphosphorylation effects in the STZ-induced hyperglycemia mouse brain.

## 2. Materials and Methods

### 2.1. Experiment Schedule

C57BL/6 mice (nine-week-old, male) were purchased from Japan SLC, Inc. (Hamamatsu, Japan). All animal experiments were performed with the approved protocol by the Animal Care and Use Committee of Matsuyama University (#9-002; 2 September, 2009). Mice were housed in a room maintained at a constant temperature of 23 ± 1 °C with 12-h light/dark cycle (lights on 8:00–20:00). Mice were given food and water ad libitum for the duration of the study [15].

Randomly, mice were divided into the following four groups (*n* = 9 each). Intraperitoneally with vehicle (saline) treatment and the oral administration of vehicle (5% dimethyl sulfoxide/H_2_O) were the control (CON) group. Intraperitoneal STZ treatment and the oral administration of the vehicle group were the STZ. STZ treatment and the oral administration of AUR (50 mg/kg; Ushio ChemiX Corp, Omaezaki, Japan) group, named AUR, and STZ treatment and the oral administration of NGI (100 mg/kg; LKT Laboratories, Inc., St. Paul, MN, USA) group, named NGI. Saline or 165 mg/kg of STZ (Wako, Osaka, Japan) was administered intraperitoneally depending on the groups on day 1. On day 8, test sample-administered groups were started to receive each sample orally once a day for 14 days. On day 22, mice were transcardially perfused with ice-cold phosphate-buffered saline (PBS) after the measurement of blood glucose, and the brains were removed.

### 2.2. Blood Glucose Measurement

To measure fasting glucose concentration on day 22, stock diets were removed from 8:00, and blood glucose was measured at 16:00. A Blood Glucose Monitoring System (Glucose Pilot; Iwai Chemicals Company, Tokyo, Japan) was used to measure blood glucose concentrations with a blood drop from the tail.

### 2.3. Immunohistochemistry and Immunofluorescence

Sagittal frozen brain sections were prepared at a thickness of 30 μm using a cryostat (CM3050S; Leica Microsystems, Heidelberger, Germany). Immunohistochemistry and immunofluorescence was performed as described in our previous study [15] with the specific primary antibodies (Table 1). Immunopositive signals in the micrographic images were quantified using ImageJ software (National Institute of Health, Bethesda, MD, USA) as described previously [15]. The positive signal densities were quantified using the “measure” tool in ImageJ software. 

### 2.4. Western Blotting Analysis

Equal amounts of protein (25 μg) of hippocampal tissues were separated on 10% sodium dodecyl sulfate-polyacrylamide gels and electroblotted onto an Immun-Blot^®^ PVDF Membrane (Bio-Rad, Hercules, CA, USA) as described in our previous study [16]. Western blotting analysis was performed with the specific primary antibodies (Table 1), and immunoreactive bands were visualized by Amersham ECL Prime Western Blotting Detection Reagent (GE Healthcare Life Sciences, Little Chalfont, UK). The band intensity was captured and measured using a LAS-3000 imaging system (Fujifilm, Tokyo, Japan).

### 2.5. Statistical Analysis

Data were analyzed by an unpaired *t*-test between two groups (CON vs STZ) and a one-factor ANOVA followed by Dunnett’s multiple comparison test among three groups (STZ vs AUR or NGI) (Prism 6; GraphPad Software, La Jolla, CA, USA).

## 3. Results

### 3.1. Suppressive Effects of AUR and NGI on Astrocyte Activation

**Figure 1:**
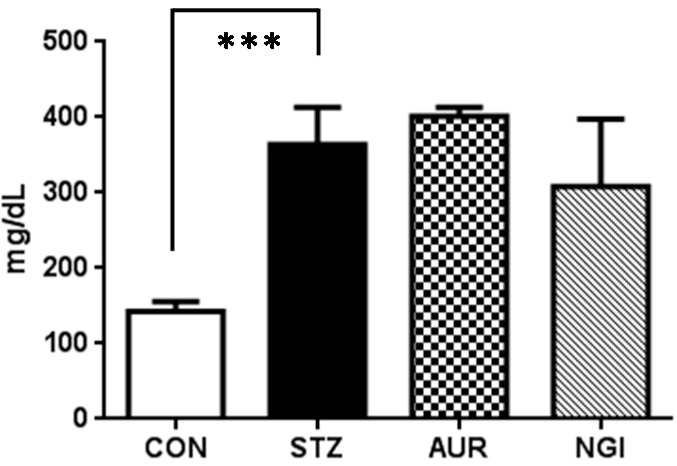
Blood glucose concentrations on Day 22. Values are means ± SEM. The symbol shows a significant difference between CON (control) and STZ (streptozotocin) (*** *p* < 0.001). AUR: auraptene; NGI: naringin.

Blood glucose concentrations were significantly elevated in the STZ group (Figure 1; *** *p* < 0.001); however, no significant changes were observed in the AUR and NGI groups.

**Figure 2:**
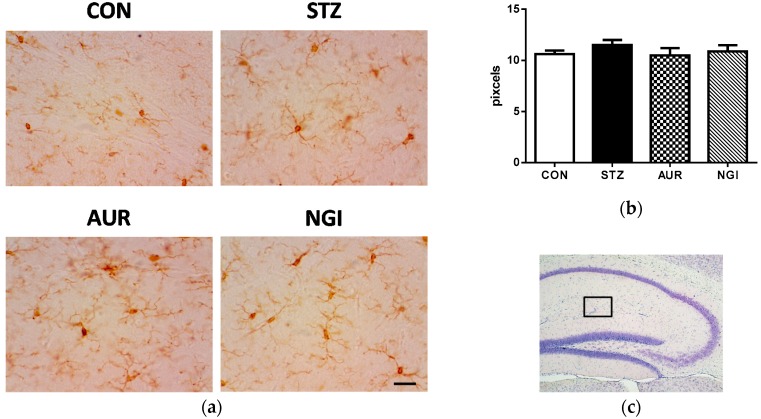
Effects of auraptene and naringin on Iba1 immunoreactivity in the hippocampus. (**a**) Sagittal sections were stained with an anti-Iba1 antibody. The scale bar shows 50 µm. (**b**) Quantitative analysis data of Iba1-positive signals using ImageJ software. (**c**) The location of the captured images. Values are means ± SEM.

**Figure 3:**
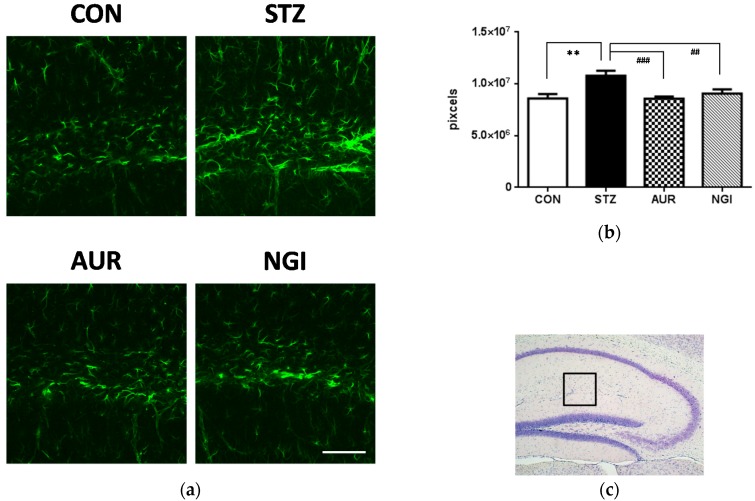
Effects of auraptene and naringin on GFAP immunoreactivity in the hippocampus. (**a**) Sagittal sections were stained with an anti-GFAP antibody. The scale bar shows 100 µm. (**b**) Quantitative analysis data of GFAP-positive signals using ImageJ software. (**c**) The location of the captured images. Values are means ± SEM. Symbols show significant differences between the following conditions: CON vs STZ (** *p* < 0.01), and STZ vs AUR or NGI (## *p* < 0.01, ### *p* < 0.001).

Hyperglycemia induces inflammation and oxidative stress in the brain [7,8], and excess glial cell activation is known to be responsible for oxidative stress and inflammatory reactions in the brain [17]. Micoglia (Iba1-positive cells) activation were not confirmed in all groups (Figure 2a,b); however, the number of reactive astrocytes that is immunostained with the GFAP antibody significantly increased in the stratum lacunosum-moleculare in the hippocampus of the STZ group (Figure 3a,b; ** *p* < 0.01). In contrast, the GFAP-positive signals were significantly suppressed in the AUR and NGI groups (Figure 3a,b; ## *p* < 0.01, ### *p* < 0.001).

**Figure 4:**
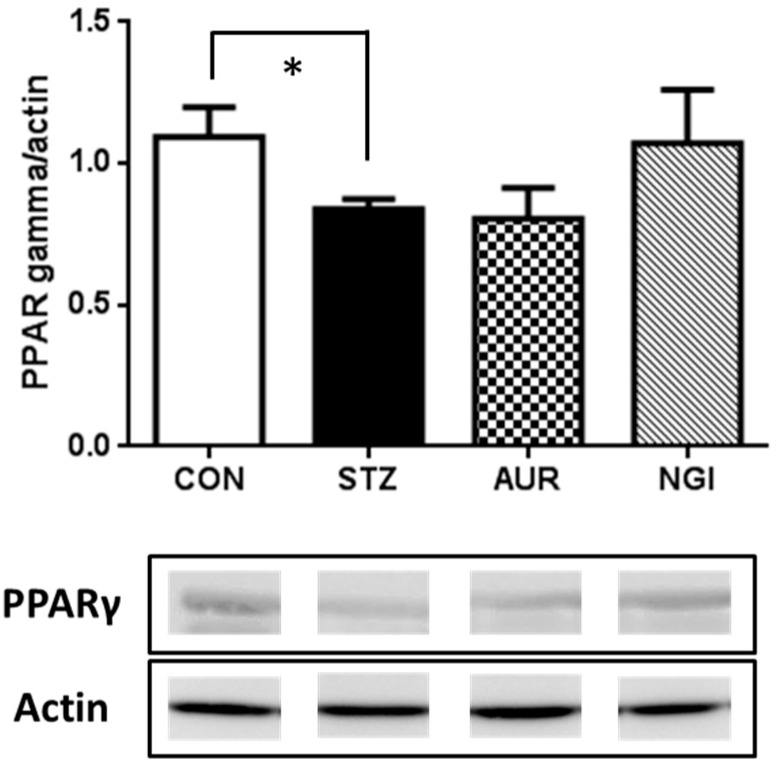
Effects of auraptene and naringin on the expression of PPARγ in the hippocampus. Values are means ± SEM. The symbol shows a significant difference between CON and STZ (* *p* < 0.05).

PPARγ regulates the cell signaling of the inflammation process and exerts anti-inflammatory effects [18], and the expression of PPARγ in the hippocampus was significantly suppressed in the STZ group (Figure 4; * *p* < 0.05); however, no significant changes were observed in the AUR and NGI groups.

### 3.2. Effects of AUR and NGI on Tau Hyperphosphorylation

**Figure 5:**
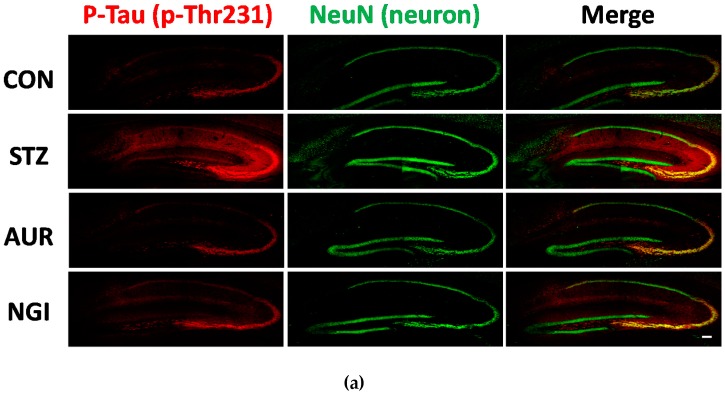

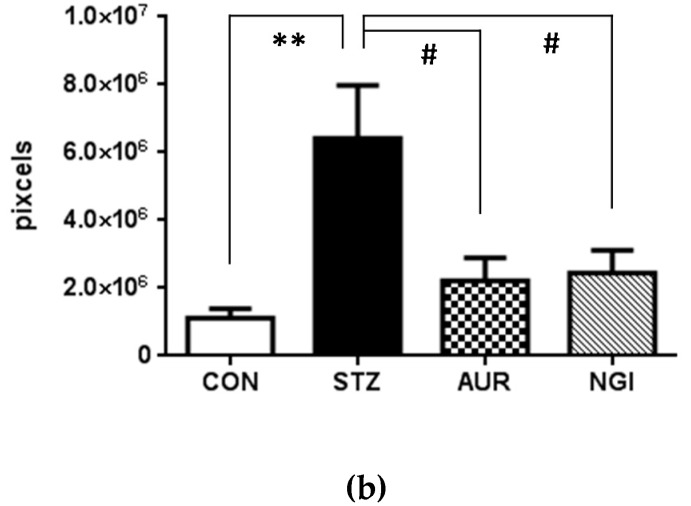
Effects of auraptene and naringin on the expression of phosphorylated Tau at 231 of threonine in the hippocampus. (**a**) Sagittal sections were stained with the anti-pThr231 (red) and NeuN (green) antibodies. The scale bar shows 100 µm. (**b**) Quantitative analysis data of pThr231-positive signals using ImageJ software. Values are means ± SEM. Symbols show significant differences between the following conditions: CON vs STZ (** *p* < 0.01), and STZ vs AUR or NGI (# *p* < 0.05).

**Figure 6:**
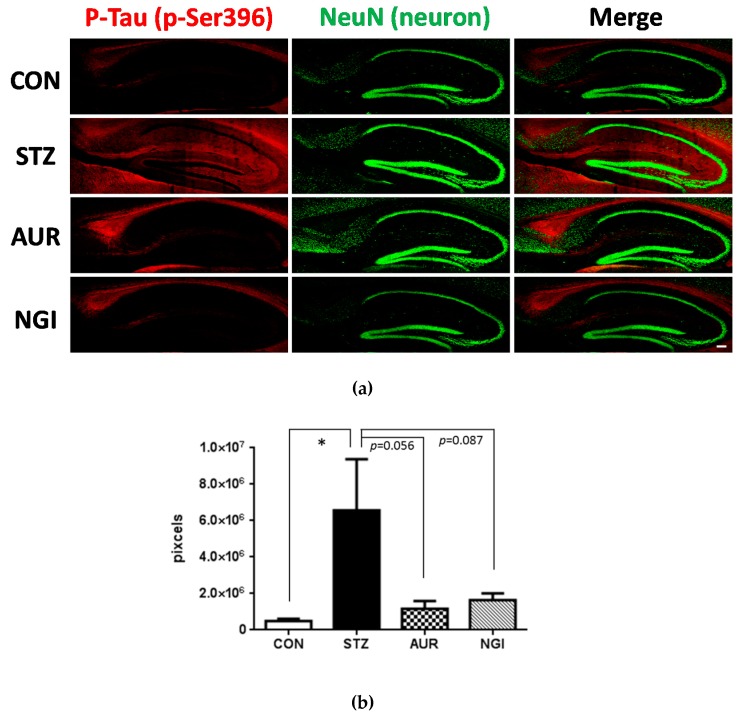
Effects of auraptene and naringin on the expression of phosphorylated Tau at 396 of serine in the hippocampus. (**a**) Sagittal sections were stained with the anti-pSer396 (red) and NeuN (green) antibodies. The scale bar shows 100 µm. (**b**) Quantitative analysis data of pSer396-positive signals using ImageJ software. Values are means ± SEM. Symbols show significant differences between CON vs STZ (* *p* < 0.05).

Tau, a microtubule-associated cytoskeletal protein, in the neuron has been understanding to relate to the molecular mechanisms for neurofibrillary tangle (NFT) formation through its multiple phosphorylative and conformational changes. It was previously shown that inflammation and oxidative stress induce tau hyperphosphorylation [19,20], and this was confirmed in STZ-injected mice brain [21]. We evaluated tau phosphorylation levels at 231 of threonine (p-Thr231) and 396 of serine (p-Ser396) in the hippocampus (Figure 5 and Figure 6). Strong positive signals for p-Thr231 were confirmed in the CA3 region pyramidal cell layer and in the stratum lacunosum-moleculare in the hippocampus. The integrated densities of the immune-positive signals were evaluated, and that was significantly higher in the STZ group (Figure 5a,b; ** *p* < 0.01); however, significant suppressive effects on its phosphorylation was observed in the AUR and NGI treated groups (Figure 5a,b; # *p* < 0.05). Similar to p-Thr231, the levels of p-Ser396 in the hippocampus were significantly higher in the STZ group than in the CON group (Figure 6a,b; * *p* < 0.05). The signals were confirmed in hippocampal mossy fibers and the stratum lacunosum-moleculare. On the other hand, the AUR and NGI treatments exerted a tendency of suppressive effects (Figure 6a,b; *p* = 0.056 and *p* = 0.087, respectively).

### 3.3. Enhancement of Neurogenesis by AUR and NGI in the Hippocampus

**Figure 7:**
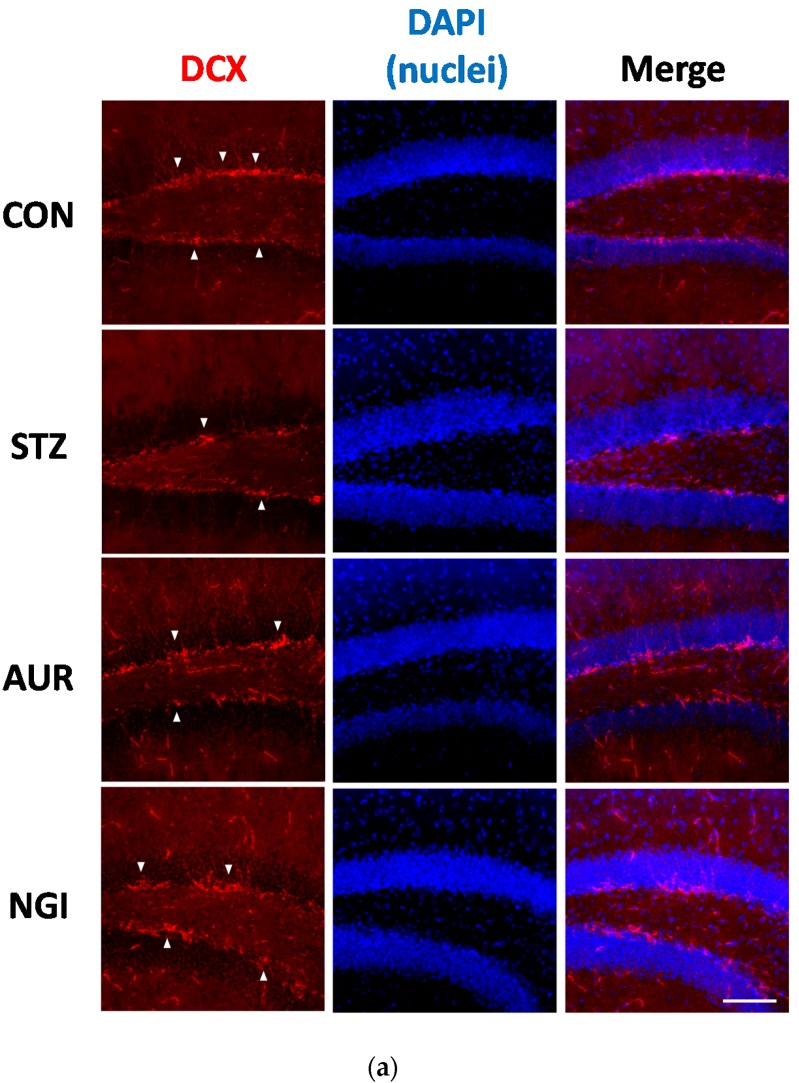

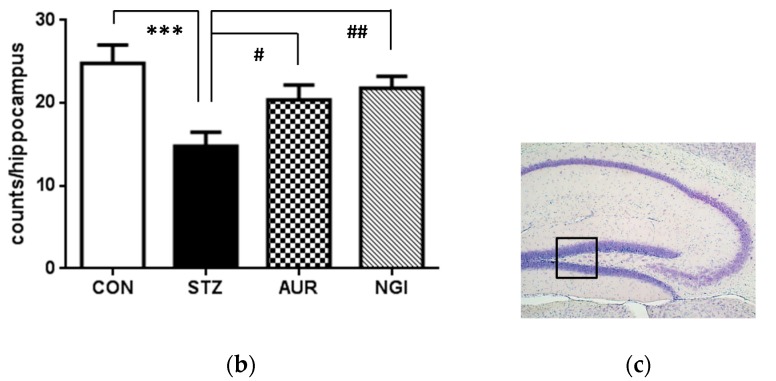
Effects of auraptene and naringin on doublecortin immunoreactivity in the hippocampus. (**a**) Sagittal sections were stained with the anti-DCX antibody (red) and DAPI (blue). The white arrowheads indicate typical DCX-positive cells in the DG (dentate gyrus). The scale bar shows 100 µm. (**b**) Counting data of DCX-positive signals in the dentate gyrus. (**c**) The location of the captured images is shown with a square in the figure. Values are means ± SEM. Symbols show significant differences between the following conditions: CON vs STZ (*** *p* < 0.001), and STZ vs AUR or NGI (# *p* < 0.05, ## *p* < 0.01).

The subgranular zone (SGZ) of the dentate gyrus (DG) is one of the areas in which neurogenesis occurs in the hippocampus [22], and adult neurogenesis is known to play an important role in learning and memory. A previous report showed that suppression of the neurogenesis in the DG was confirmed, following a STZ administration [23]. Figure 6 shows the immunoreactivity of DCX, a marker for immature neurons, in the SGZ. DCX-positive cells were suppressibility observed in the STZ group, but markedly higher number in the AUR and NGI groups (Figure 7a). We manually counted the DCX-positive neurons, with checking the soma and nucleus, in the SGZ under the fluorescence microscopy and evaluated the number of DCX-positive newborn neurons in the SGZ. The numbers of the STZ group showed significantly suppressed expression (Figure 7b; *** *p* < 0.001); in contrast, the AUR and NGI groups ameliorated this suppression (Figure 7b; # *p* < 0.05, ## *p* < 0.01).

## 4. Discussion

Hyperglycemia induces inflammation and oxidative stress in the central nervous system [24], and diabetes is currently regarded as one of the risk factors for dementia, such as AD and vascular dementia [2,3,4]. The AD brain is frequently associated with severe inflammation, oxidative stress, neuronal dysfunction, amyloid-beta accumulation, tau hyperphosphorylation, and memory impairment [5]. Several studies indicate inflammation and oxidative stress increased tau hyperphosphorylation of neurons in the brain [19,20]; therefore, our primary focus was to clarify the anti-inflammatory and tau hyperphosphorylation suppression effects of AUR and NGI in a hyperglycemia model.

STZ-treated mice showed significantly elevated blood glucose concentrations, whereas suppressive effects were not observed in the NGI and AUR groups (Figure 1). Previous studies reported that the administration of 100 mg/kg of NGI suppressed blood glucose concentrations in STZ-treated rats, or 0.2% of AUR administration resulted in suppressive effects on high-fat diet-induced obese mice, respectively [25,26]. These findings suggested that NGI and AUR have the potential to reduce blood glucose concentrations in several diabetic models. However, treatments with NGI and AUR did not reduce blood glucose concentrations in our experiment.

In hyperglycemia models, astrocyte activation is related to immune response in the brain [27,28]. Treatments with AUR and NGI suppressed astrocyte activation in this experiment (Figure 3), and we previously demonstrated that the activation of astrocytes was inhibited by the AUR treatment in a transient global cerebral ischemic mouse model [10]. Strong microglial activation, also an immune response cell in the brain, was not observed in the STZ group (Figure 2) in this experiment, whereas astrocytes were detected. Microglia is activated earlier than astrocytes in a manner that is dependent on disease conditions [29]; therefore, we considered strong microglial activation to only occur in the early phase of the STZ treatment. In fact, we previously demonstrated that microglial activation was confirmed one week after the administration of STZ [30]; however, the time point of sacrifice in this experiment was two weeks after the STZ treatment. PPARγ is an important target in diabetes therapy, and regulates the cell signaling of the inflammation process [18]. AUR and NGI did not significantly affect the protein expression of PPARγ in the hippocampus in this experiment (Figure 4); on the other hand, a previous study reported that NGI ameliorated cognitive deficits via oxidative stress and proinflammatory factor suppression, and activated the protein expression of PPAR in the hippocampus of an STZ-injected rat model [25]. AUR also activates PPARγ in adipocytes [31]. Collectively, these findings in our experiment indicated that AUR and NGI exerted anti-inflammatory effects by suppressing the activation of astrocytes in the hippocampus, though we still have to do further experiments to see the detail mechanism.

Increases in inflammation and oxidative stress induce the hyperphosphorylated tau protein in neurons and this is enhanced in the hyperglycemic brain [19,20]. The microtubule function of neurons is maintained by the phosphorylation of the tau protein, and the regulation of kinases (including CDK-5 and GSK-3β) and phosphatases (such as PP2A) are very important; however, the hyperphosphorylation of tau induces microtubule dysfunction, leading to the formation of NFT, which is often observed in the AD brain [32]. In tau protein, several strong phosphorylation sites have been identified, such as Thr231 and Ser396, in hyperglycemic animal brains [21,33], and oxidative stress and inflammation may induce a kinase and phosphatase imbalance [34]. We focused on phosphorylation sites in Thr231 and Ser396 in the present study, and AUR and NGI treatment exerted suppressive effects on tau phosphorylation in the hippocampus in STZ-treated mice (Figure 5 and Figure 6). Neurogenesis in the SGZ of the DG in the hippocampus is of particular importance for hippocampal-dependent memory function [35,36]. It is suppressed by a number of conditions, including depression, AD, and aging; in addition, hyperglycemia has also been shown to suppress neurogenesis [37,38]. Staining for neurogenesis with DCX, the AUR and NGI treatments enhanced its expression (Figure 7). Inhibitory effects of AUR and NGI on tau hyperphosphorylation and suppression of neurogenesis were our new findings.

## 5. Conclusions

When AUR and NGI were administered to STZ-injected hyperglycemia mice, they (1) suppressed astroglial activation; (2) diminished tau phosphorylation; and (3) stimulated neurogenesis in the SGZ of the DG in the hippocampus. These results suggest that AUR and NGI, citrus-related compounds, exert anti-inflammatory and antioxidative effect against hyperglycemia-induced changes in the brain, and have potential as novel neuroprotective agents obtained from food materials.

## Figures and Tables

**Table 1:** Summary of primary antibodies used for immunohistochemiatry, immunofluorescence and western blotting analysis.

Antibody	Epitope Protein/Amino Acids	Host	Dilution	Resource
Iba1	ionized calcium-binding adaptor molecule 1	rabbit	1:1000	Wako, Osaka, Japan
GFAP	glial fibrillary acidic protein	mouse	1:200	Sigma-Aldrich, St. Louis, MO, USA
p-Thr231	phosphorylated-tau Threonine 231	rabbit	1:1000	AnaSpec, Fremont, CA, USA
p-Ser396	phosphorylated-tau Serine 396	rabbit	1:1000	AnaSpec
NeuN	neuronal nuclei	mouse	1:200	Millipore, Temecula, CA, USA
DCX	doublecortin	goat	1:50	Santa Cruz Biotechnology, Santa Cruz, CA, USA
PPARγ	peroxisome proliferator-activated receptor-gamma	rabbit	1:1000	Abcam, Cambridge, UK
Actin	actin	rabbit	1:1000	Sigma-Aldrich
